# Survey of influenza and other respiratory viruses diagnostic testing in US hospitals, 2012–2013

**DOI:** 10.1111/irv.12355

**Published:** 2016-01-29

**Authors:** Su Su, Alicia M. Fry, Pam Daily Kirley, Deborah Aragon, Kimberly Yousey‐Hindes, James Meek, Kyle Openo, Oluwakemi Oni, Ruta Sharangpani, Craig Morin, Gary Hollick, Krista Lung, Matt Laidler, Mary Lou Lindegren, William Schaffner, Annette Atkinson, Sandra S. Chaves

**Affiliations:** ^1^ Influenza DivisionCenters for Disease Control and PreventionAtlantaGAUSA; ^2^ Atlanta Research and Education FoundationAtlantaGAUSA; ^3^ California Emerging Infections ProgramOaklandCAUSA; ^4^ Colorado Department of Public Health and EnvironmentDenverCOUSA; ^5^ Connecticut Emerging Infectious ProgramYale School of Public HealthNew HavenCTUSA; ^6^ Georgia Emerging Infections Program and the Atlanta Research and Education FoundationAtlantaGAUSA; ^7^ Iowa Department of Public HealthDes MoinesIAUSA; ^8^ Michigan Department of Health and Human ServicesLansingMIUSA; ^9^ Minnesota Department of HealthSt. PaulMNUSA; ^10^ University of Rochester Center for Community HealthMinneapolisMNUSA; ^11^ Ohio Department of HealthColumbusOHUSA; ^12^ Oregon Public Health DivisionPortlandORUSA; ^13^ Vanderbilt University School of MedicineNashvilleTNUSA; ^14^ Utah Department of HealthSalt lake cityUTUSA

**Keywords:** EIP, FluSurv‐NET, influenza, laboratory capacity, respiratory viruses

## Abstract

**Background:**

Little is known about laboratory capacity to routinely diagnose influenza and other respiratory viruses at clinical laboratories and hospitals.

**Aims:**

We sought to assess diagnostic practices for influenza and other respiratory virus in a survey of hospitals and laboratories participating in the US Influenza Hospitalization Surveillance Network in 2012–2013.

**Materials and Methods:**

All hospitals and their associated laboratories participating in the Influenza Hospitalization Surveillance Network (FluSurv‐NET) were included in this evaluation. The network covers more than 80 counties in 15 states, CA, CO, CT, GA, MD, MN, NM, NY, OR, TN, IA, MI, OH, RI, and UT, with a catchment population of ~28 million people. We administered a standardized questionnaire to key personnel, including infection control practitioners and laboratory departments, at each hospital through telephone interviews.

**Results:**

Of the 240 participating laboratories, 67% relied only on commercially available rapid influenza diagnostic tests to diagnose influenza. Few reported the availability of molecular diagnostic assays for detection of influenza (26%) and other viral pathogens (≤20%) in hospitals and commercial laboratories.

**Conclusion:**

Reliance on insensitive assays to detect influenza may detract from optimal clinical management of influenza infections in hospitals.

## Background

The accuracy of diagnosing influenza virus infections on the basis of symptoms alone is limited, as symptoms from illnesses caused by other respiratory pathogens overlap considerably.^1^, ^2^ Early and accurate identification of influenza can reduce the inappropriate use of antibiotics and inform treatment decisions regarding the use of influenza antiviral agents.^3^, ^4^ Several influenza diagnostic assays are commercially available and include rapid influenza diagnostic tests (RIDTs), immunoassays that identify viral antigens in respiratory specimens, and more recently, molecular based assays that detect viral nucleic acid in respiratory specimens.^5^ Prior to the 2009 influenza pandemic, the U.S. public health infrastructure's capacity to quickly and accurately detect influenza viruses, including novel influenza A viruses, was established. This capacity was dependent upon building and maintaining molecular diagnostic capacity for influenza viruses among public health laboratories in the United States.^6^, ^7^ This greatly strengthened molecular diagnostic capacity for influenza among public health laboratories in the United States. However, less is known about laboratory capacity to routinely diagnose influenza and other respiratory viruses at clinical laboratories and hospitals. Our objective was to describe influenza and other respiratory virus diagnostic practices and routine laboratory testing capacity from a sample of laboratories serving hospitals in the United States.

## Methods

All hospitals and their associated laboratories (at hospital and external contracting laboratories) participating in the Influenza Hospitalization Surveillance Network (FluSurv‐NET) were included in this evaluation.^8^, ^9^ The network covers more than 80 counties in 15 states, CA, CO, CT, GA, MD, MN, NM, NY, OR, TN, IA, MI, OH, RI, and UT, with a catchment population of ~28 million people. The surveillance is based on identification of positive laboratory results for influenza. However, whether or not a hospitalized patient is tested for influenza is dependent upon the clinical practices of the team caring for each individual patient. During 2012–2013, participating hospital laboratories were asked to voluntarily send influenza A specimens to state public health laboratories for subtyping. Some network sites also asked hospital laboratories to send RIDT‐negative specimens to public health laboratories to improve case numbers.

During the 2012–2013 influenza season (October–April), we administered a standardized questionnaire to key personnel at each hospital through telephone interviews. The first part of the questionnaire, administered to infection control practitioners, assessed characteristics of hospitals, policies in place for testing patients with acute respiratory illness at the emergency department (ED), and availability of influenza point‐of‐care (POC) tests which are designed to be used at or near site where the patient is located and allows timely diagnosis. The second part of the questionnaire, administered to the head of laboratory departments or their designees, collected information on diagnostic capacity for influenza and other respiratory viruses in the laboratories. Questions included type of laboratory tests available, specimen type acceptable for testing, turnaround time for molecular diagnostic tests, and testing algorithm for confirmation of initial results. We compared laboratory capacity for influenza testing with results collected from a similar survey conducted in a subset of the current network sites (CA, CO, CT, GA, MD, MN, NM, NY, OR, TN) during pre‐pandemic era (in 2006–2007 influenza season) (S.C. Chaves, unpublished) using chi‐square test. The analyses were performed in SAS 9.3 (SAS Institute, Cary, NC, USA).

## Results

### Characteristics of hospitals

Most (267/316, 84%) participating hospitals in the FluSurv‐NET catchment area responded to the survey; 206 (78%) were located in urban areas (i.e. population density ≥ 2000 people per square mile), whereas the remainder were located in suburban or rural areas. Approximately half (*n* = 129) of all hospitals had fewer than 200 beds. The hospitals represented a variety of practices including 210 general hospitals, 16 children's hospitals, 12 Veteran's Affairs hospitals and 7 other specialty hospitals. Fifty‐three (20%) of the hospitals were affiliated with academic institutions and 220 (83%) were equipped with a general ED.

The majority of the hospitals (80%) did not have a policy in place to systematically test patients with acute respiratory infections (ARI) presenting to the ED during influenza seasons for influenza. All hospitals that had a policy in place for testing patients with ARI were located in urban/suburban areas. Having a testing policy was not associated with hospital size or academic affiliation. Most of these hospitals applied the testing policy to both adults and children (42/52).

Sixty (22%) hospitals performed RIDTs during influenza season directly in the ED or clinic (i.e. ‘point‐of‐care testing’): 55/60 in the ED, 20 of 60 in outpatient clinics, and 31 of 60 in inpatient wards. Most of these hospitals were urban (45/60), non‐academic affiliated (49/60) and with less than 200 beds (40/60). There were no difference in the availability of POC testing and a hospital policy to test patients for influenza during influenza season (20% versus 20%, *P* = 0·79).

### Laboratory capacity for influenza diagnostic testing

**Figure 1:**
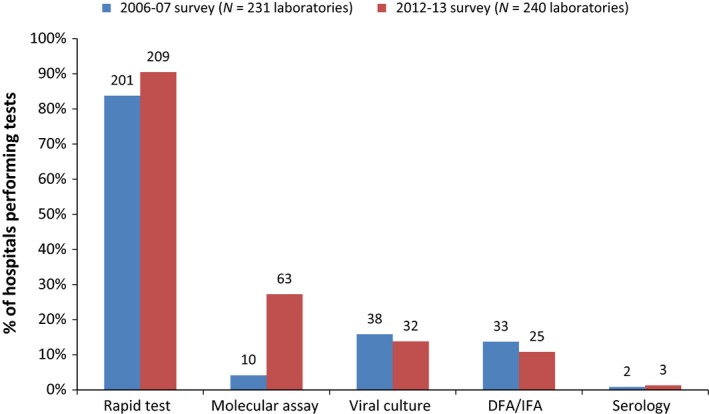
Comparison of types of influenza diagnostic tests performed in clinical laboratories serving hospitals during the 2006–2007 and 2012–2013 influenza season.

Two hundred and twenty‐nine hospital laboratories and 11 independent laboratories (e.g. reference, commercial, physician's office, satellite clinic) responded to the survey. All laboratories had at least one influenza diagnostic assay available. RIDTs were the most frequently used (87%), followed by molecular assays (26%), viral culture (13%), direct or indirect fluorescent antibody assay (DFA/IFA) (10%), and serology (1%). RIDT was the only diagnostic test available for influenza in 67% of the laboratories. Compared to results from the survey conducted in 2006–2007, the proportion of laboratories reporting the use of molecular assays increased significantly during the 2012–2013 season (4% versus 26% respectively, *P* < 0·001), while the use of viral culture and DFA/IFA slightly decreased (Figure 1). Among 63 laboratories with reverse transcription polymerase chain reaction (RT‐PCR) capacity, 8% developed their own influenza RT‐PCR assay in house and 60% were able to subtype influenza A viruses. Most of these laboratories (57/63) were capable of providing RT‐PCR test results within 1 day, among which 60% (34/57) ran tests immediately or as needed. The other six laboratories performed testing at various frequencies within a week.

### Influenza testing algorithms

**Figure 2:**
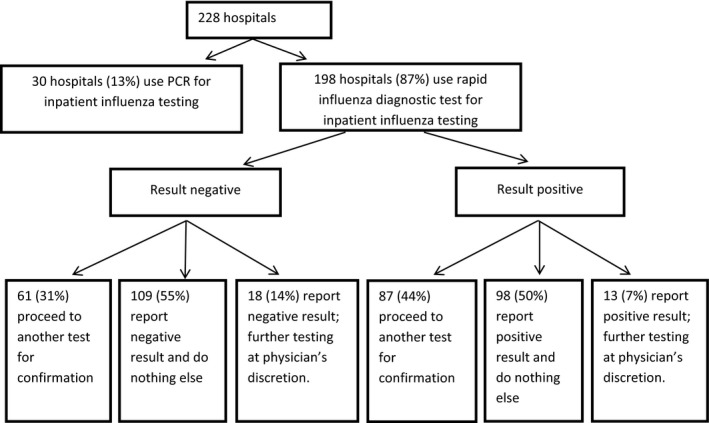
Description of influenza testing practices in hospital laboratories, 2012–2013. Note: Hospitals with missing data are excluded. Confirmatory testing could be performed at the hospital laboratory or at public health laboratories. Participating hospital laboratories were asked to send influenza A specimens to state public health laboratories for subtyping. Some network sites asked laboratories to send RIDT‐negative specimens to public health laboratories.

Among 228 local hospital laboratories with available data, 30 (13%) used molecular testing as the only technique for inpatient influenza testing (Figure 2). Among 198 laboratories that performed RIDT as their first choice for an influenza diagnostic, ~50% reported both positive and negative results back to the physician as final, without proceeding with a confirmatory test. Laboratories were more likely to send positive RIDT results for confirmation than negative RIDT results (44% versus 31%); this reflects participation in FluSurv‐NET surveillance for influenza A virus subtypes. Most of the confirmatory testing was sent to and performed at the corresponding state public health reference laboratory.

### Testing capacity for respiratory viruses other than influenza

Overall, 172 of 240 (72%) of the laboratories were able to perform some kind of testing for other respiratory viruses (Table 1); 55 of 172 (32%) offered a molecular multiviral panel which often included influenza virus, respiratory syncytial virus (RSV), parainfluenza viruses, human metapneumovirus (hMPV), human rhinoviruses, and adenoviruses. Among the 172 laboratories performing tests for respiratory viruses other than influenza, all were able to test for RSV, 30% for adenovirus, 29% for parainfluenza viruses, 18% for hMPV, 13% for rhinovirus, and 3% for coronavirus. Rapid RSV diagnostic test was more frequently used (62%) for RSV testing than molecular assay (20%). For other respiratory viruses, the most common test available was a molecular assay (including Luminex, FilmArray, nucleic acid test (NAT), and RT‐PCR assays).

**Table 1:** Laboratory capacity to perform diagnostic tests for respiratory viruses other than influenza at 240 hospital clinical laboratories

Virus	Number of laboratories performing test	Diagnostic tests used
Viruses other than influenza	172/240 (72%)	
Respiratory syncytial virus (RSV)	172/240 (72%)	Rapid test (62%); molecular assay (20%); DFA/IFA (9%); culture analysis (9%)
Adenovirus	52/240 (22%)	Molecular assay (53%); culture analysis (27%); DFA (20%)
Parainfluenza viruses	50/240 (21%)	Molecular assay (50%); culture analysis (25%); DFA/IFA (25%)
Human metapneumovirus	31/240 (13%)	Molecular assay (80%); DFA (16%); culture analysis (4%)
Rhinovirus	22/240 (9%)	Molecular assay (77%); culture analysis (23%)
Coronavirus Enterovirus	5/240 (2%)	Unspecified by reporting laboratories

Assays included as molecular assays: Luminex, FilmArray, nucleic acid test (NAT), and reverse transcription polymerase chain reaction (RT‐PCR).

## Discussion

We demonstrated predominant use of rapid diagnostic tests for influenza and RSV during 2012–2013 among hospitals and laboratories from 15 states. While the use of molecular diagnostic assays for detection of influenza virus infection at hospital and associated commercial laboratories increased modestly since 2006–2007, the availability of influenza molecular diagnostics for clinical care at hospitals was still limited; only 26% of hospital laboratories reported availability of molecular diagnostic assays. Laboratory diagnostics for respiratory viruses other than influenza and RSV were uncommonly available. Also, a minority of hospitals included in our survey had policies in place to systematically test patients with acute respiratory infections (ARI) seen in the ED with influenza diagnostics during influenza season. As the number and type of commercially available laboratory diagnostics for respiratory pathogens evolve, an updated survey is warranted.

We showed that in most (67%) hospitals included in FluSurv‐NET, a U.S. population‐based surveillance network involving 15 states, RIDT was the clinician's primary or only option for influenza diagnostic testing. RIDTs are easy to use and have rapid turnaround time for results. However, RIDTs have been shown to have suboptimal sensitivity (40–70%) compared to RT‐PCR or viral culture.^10^, ^11^, ^12^, ^13^ Several studies suggest that healthcare providers are more likely to prescribe antiviral drugs with a positive RIDTs.^13^, ^14^ Therefore, a false‐negative result may affect clinicians’ decision to provide appropriate treatment and infection control among patients with influenza. Given the suboptimal sensitivity of RIDT, patient management should not depend upon a positive RIDT result. If patient management depends upon a diagnosis (or rule out) of influenza, additional confirmatory test with a more sensitive assay is warranted and the initiation of antiviral treatment and implementation of infection control should be started empirically and not delayed while awaiting confirmation.^15^ In our survey, only 31% of laboratories had protocols for confirmatory testing with a more sensitive assay following a negative rapid test result. This may reflect the limited usefulness of receiving testing results late in clinical care. Also, in our survey, the laboratories confirming RIDT test results reflects shipment of specimens to state public health laboratories as part of FluSurv‐NET surveillance, in addition to clinician practice. New molecular assays may improve the accuracy of influenza diagnosis, but timeliness and cost of these new assays could affect future use.

Our survey has some limitations. The survey was completed in 2012–2013 and may not be representative of laboratories in more recent seasons, especially in light of expanding commercially available molecular assays, and may not be representative of all hospital laboratories in United States. Hospitals in the FluSurv‐NET catchment area have regular correspondence with state health department and may have better access to laboratory diagnostics than elsewhere, and physicians in these settings may be more aware of the importance of early diagnosis of influenza and other viral pathogens. Nonetheless, it provided an overview of local laboratory capacity in the nation as it reflected over 260 hospitals of varying characteristics and 240 supporting laboratories. Finally, the hospital laboratories that did not respond to our survey provide few cases to FluSurv‐NET and may be different from the participating laboratories.

In conclusion, our survey suggests that most hospitals in the United States in 2012–2013 were heavily dependent on RIDT to diagnose influenza. Reliance upon an insensitive assay may compromise patient treatment and infection control. Increasing use of molecular assays for respiratory viruses may improve patient care and surveillance for respiratory viruses.

## Disclaimer and financial support

The Influenza Hospitalization Surveillance Network (FluSurv‐NET) is a collaboration of state health departments, academic institutions and local partners and is funded by the Centers for Disease Control and Prevention (CDC). This publication was supported in part by Cooperative Agreement number CDC‐RFA‐CK12‐1202 and 5U38HM000414 from CDC. Its contents are solely the responsibility of the authors and do not necessarily represent the official views of the Centers for Disease Control and Prevention, US Department of Health and Human Services.
